# Acetylacetone photodynamics at a seeded free-electron laser

**DOI:** 10.1038/s41467-017-02478-0

**Published:** 2018-01-04

**Authors:** R. J. Squibb, M. Sapunar, A. Ponzi, R. Richter, A. Kivimäki, O. Plekan, P. Finetti, N. Sisourat, V. Zhaunerchyk, T. Marchenko, L. Journel, R. Guillemin, R. Cucini, M. Coreno, C. Grazioli, M. Di Fraia, C. Callegari, K. C. Prince, P. Decleva, M. Simon, J. H. D. Eland, N. Došlić, R. Feifel, M. N. Piancastelli

**Affiliations:** 1 Department of Physics, University of Gothenburg, Origovägen 6B, SE-412 96 Gothenburg, Sweden; 2 Institut Ruđer Bošković, Bijenička cesta 54, 10000 Zagreb, Croatia; 3 Elettra-Sincrotrone Trieste, Strada Statale 14-km 163.5, 34149 Basovizza, Trieste, Italy; 4 Consiglio Nazionale delle Ricerche-Istituto Officina dei Materiali, 34149 Trieste, Italy; 5 Sorbonne Universités, UPMC Univ Paris 06, CNRS, UMR 7614, Laboratoire de Chimie Physique-Matière et Rayonnement, 75005 Paris Cedex 05, France; 6 Consiglio Nazionale delle Ricerche – Istituto di Struttura della Materia, LD2 unit, 34149 Trieste, Italy; 7 Molecular Model Discovery Laboratory, Department of Chemistry and Biotechnology, Swinburne University of Technology, Melbourne, VIC 3122 Australia; 8 Dipartimento di Scienze Chimiche e Farmaceutiche, Universitá di Trieste, 34127 Trieste, Italy; 9 Department of Chemistry, Physical and Theoretical Chemistry Laboratory, Oxford University, South Parks Road, Oxford, OX1 3QZ UK; 10 Department of Physics and Astronomy, Uppsala University, SE-751 20 Uppsala, Sweden

## Abstract

The first steps in photochemical processes, such as photosynthesis or animal vision, involve changes in electronic and geometric structure on extremely short time scales. Time-resolved photoelectron spectroscopy is a natural way to measure such changes, but has been hindered hitherto by limitations of available pulsed light sources in the vacuum-ultraviolet and soft X-ray spectral region, which have insufficient resolution in time and energy simultaneously. The unique combination of intensity, energy resolution, and femtosecond pulse duration of the FERMI-seeded free-electron laser can now provide exceptionally detailed information on photoexcitation–deexcitation and fragmentation in pump-probe experiments on the 50-femtosecond time scale. For the prototypical system acetylacetone we report here electron spectra measured as a function of time delay with enough spectral and time resolution to follow several photoexcited species through well-characterized individual steps, interpreted using state-of-the-art static and dynamics calculations. These results open the way for investigations of photochemical processes in unprecedented detail.

## Abstract (web summary)

The first steps in photochemical processes involve changes in electronic and geometric structure on extremely short timescales. Here, the authors report femtosecond dynamics in prototypical acetylacetone, by pump-probe photoexcitation-photoemission experiments and static and dynamics calculations.

## Introduction

It is a long sought-after goal to follow the dynamics of photoexcited molecular species on very short time scales using such tools as free-electron lasers (FELs) or high-harmonic generation (HHG). A colorful description of the ultimate objective is to call it the making of a “molecular movie”. Various attempts to characterize molecular dynamical processes on ultrashort time scales have been made at FELs, mostly with structural techniques such as photoelectron diffraction^1^, and X-ray diffraction/scattering^2–4^. Such methods require complex data manipulations and are insensitive to subtle differences in electronic structure, which albeit small can be vital to the process. A promising way to characterize the full electronic dynamics would be to look at valence photoelectron spectra in combination with ion yield spectra, to identify photochemical reaction pathways. Valence photoelectron spectra are directly informative of the electronic structure of the investigated species and can reveal variations in response to external parameters such as temperature change or photoexcitation. To observe the first stages of a chemical process using photoelectron spectra, picosecond (ps) or femtosecond (fs) time resolution is needed and the most suitable light sources to employ are FELs or HHG. Up to now this relatively simple approach has been hindered by the facts that at FELs the photon energy jitter is generally too large to give sufficiently resolved spectra without heavy data treatment and/or averaging over several time delays^5,6^, and with simple HHG the resolution is masked by the simultaneous presence of multiple harmonics. If harmonic filtering is employed, the resulting intensity is too low to follow subtle changes.

**Fig. 1:**
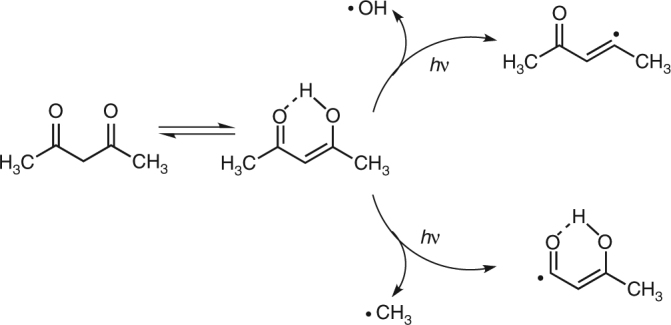
Chemical formulas for the two tautomeric forms and possible fragmentation pathway

A recent major breakthrough in this direction is represented by the newly built FERMI FEL at the Elettra facility, Trieste, Italy^7^. This seeded source provides pulses with negligible photon energy jitter, so photoemission spectra can be obtained with enough resolution to characterize very precisely ionization from different electronic states. The seeding scheme also provides an IR pulse which is very accurately synchronized to the FEL pulse, because it is derived from the Ti:Sa laser that drives the seed chain^8–10^. This IR pulse can be conditioned (change of delay, polarization, intensity; frequency doubling/tripling), and is conventionally named seed laser for users (SLU). Using the FERMI-seeded source in combination with the third harmonic of the SLU, 261 nm (4.75 eV), we have performed pump-probe experiments on a prototypical system, acetylacetone, which is a stable molecule used as solvent or as chelating agent^11,12^ with potential environmental and medical applications^13^. The stable form in the gas phase at room temperature is the enol form stabilized by the intramolecular hydrogen bond; it can convert to the tautomeric keto form (see Fig. 1 for chemical formulas and possible fragmentation pathways) by proton transfer as a result of thermal activation or photoexcitation^14–17^. The photoexcitation/decay pattern of acetylacetone in the gas phase upon irradiation with a 266 nm wavelength has been reported in the literature^17–19^. Experimental evidence on the short time evolution remain however fragmentary, and conclusions rather tentative. A clearer picture was proposed by a theoretical study^17^. The molecule in the enolic form is initially promoted to the Franck–Condon (FC) region of the S_2_ (ππ*) singlet state by 266 nm excitation. Decay to the S_1_ (nπ*) state takes place in a short period by vibronic interaction, and finally the molecule relaxes to the T_1_ (ππ*) triplet state by spin–orbit coupling (SOC). Rotational isomerization involving a breakage of the intramolecular O–H–O hydrogen bond and conversion of the C=C double bond to a single bond can then proceed from this state^16,17^ leading to the keto form. A competitive process of OH elimination has also been shown to take place^18^ from the lowest triplet T_1_ (ππ*) state, with a time constant of 247 ± 43 ps after initial excitation to the singlet S_2_ (ππ*) state. Another recent work^20^ reports results on acetylacetone in different solvents, employing the same pump and transient spectroscopy as the main investigating tool. The excitation to the S_2_ state and subsequent decay to the S_1_ state are described, but the following step is assigned to the formation of a reaction product, namely a nonchelate enol, by rotamerization, which is concluded to be the dominant mechanism in solution.

Here, the main goal of our experiment is to follow the dynamics of the excited species in much greater detail than achieved previously, by exploiting the advanced characteristics of the FERMI-seeded source^7^, in particular the opportunity to obtain easy-to-read photoelectron spectra with good resolution and high statistics. We record valence electron and ion spectra with a magnetic bottle spectrometer^21–23^ on the LDM (low density matter) beam line^24,25^, after pumping with the third harmonics of the SLU at 261 nm (estimated pulse duration 120–170 fs, energy per pulse up to 55 μJ) and probing with the fourth harmonic of FERMI at 19.23 eV (pulse duration ~100 fs, energy per pulse 40–60 μJ). The reported literature value for the excitation to the S_2_ (ππ*) state is 266 nm, but the absorption peak is wide (ref. ^17^ and references therein), so that our pump induces the same transition. The negative values in the pump-probe delay scales in some of the figures represent FEL-only induced processes, recorded for reference purpose. The time evolution was followed up to 1.5 ps pump-probe delay with 50-fs steps and to 200 ps with larger steps. A clear picture of the evolution of the system is reached, showing that the photoexcitation to the S_2_ (ππ*) (bright) state is followed by a conical intersection (CI) connecting with the S_1_ (nπ*) (dark) state, and then the T_1_ (ππ*) state is reached through ultrafast S_1_ (nπ*)/T_2_ (nπ*) crossing which is immediately followed by internal conversion to T_1_ (ππ*). A minor pathway leading back to the ground state is also identified. Observed fragmentation yielding CH_*x*_ species is related to the onset of the T_1_ (ππ*) state formation. We believe this approach based on high-resolution valence spectra backed by high-level calculations is the ultimate way to shed light on fundamental, basic photo processes such as photosynthesis, photovoltaic energy production, and vision.

## Results

### General remarks

A great asset of the experimental setup is that by simple changes of parameters it is possible to detect either electrons or ions with the same spectrometer and under exactly the same conditions in terms of pump-probe photon energies and delays. Let us note that the seed laser wavelength of the FEL, *λ*_seed_, is independent of that of the SLU, and can be tuned between 230 and 260 nm^7^ in standard configuration. By changing transport mirrors, and making other modifications, it is also possible to cover the range 290 to 360 nm. The wavelength of the FEL, *λ*_FEL_, is then determined by setting its undulators at the desired harmonic *n* of *λ*_seed_; for technical convenience we chose *λ*_seed_ = 257.85 nm and set the undulators to *n* = 4, hence *λ*_FEL_ = 64.46 nm. The residual time jitter between the FEL and the SLU is estimated at 6 fs^26,27^. Negative time delays in the horizontal scales for both ion and photoelectron spectra indicate delays where FEL ionization comes before the UV excitation pulse, i.e., FEL-only measurements. We have also run SLU-only spectra, to rule out the possibility of multiphoton processes induced by the pump. Two photons are sufficient to ionize the molecule, and ionization has been found to be negligible.

### Ion spectra

**Fig. 2:**
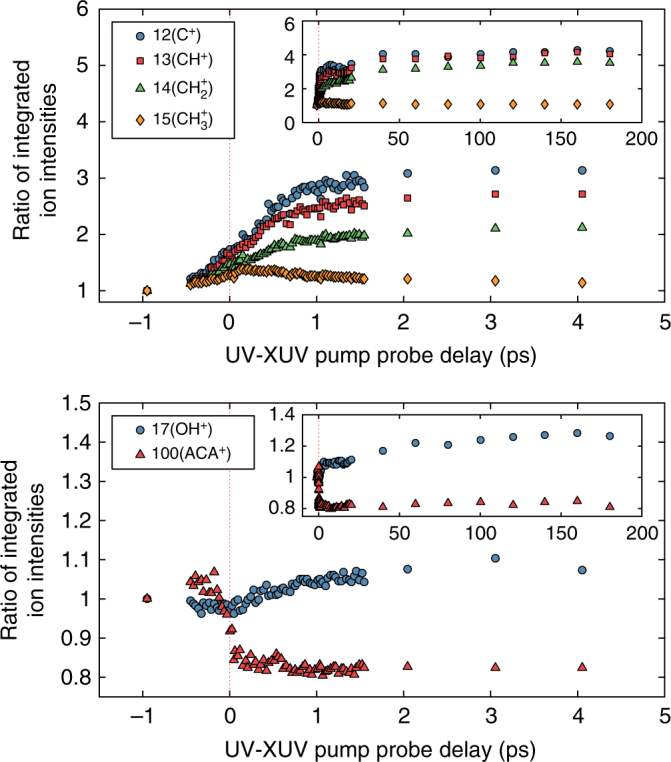
Ratios of integrated ion intensities. Top of panel: ratio of integrated ion intensities at a given delay to the integrated ion intensity at −1 ps (FEL only) for the fragments CH_*x*_^+^ in the range 0–5 ps. Inset: same in the enlarged scale 0–200 ps. Bottom of panel: ratio of integrated ion intensities at a given delay to the integrated ion intensity at −1 ps (FEL only) for the fragments OH^+^ and the parent ion as a function of pump-probe delay in the range 0–5 ps. Inset: same in the extended delay range 0–200 ps

The ion yield spectra for the most relevant ions are shown in Fig. 2 as functions of the pump-probe delay on time scales up to 5 and 200 ps. For each yield, the ratio of the integrated peak intensity between the initial value with FEL only and the values as a function of the pump-probe time delay are shown, to underline the relative variations. The dominant process revealed by the ion spectra is a fragmentation leading to production of the CH_*x*_^+^ ion family (*x* = 3, 2, 1, 0) within about 1 ps. All these ions could be produced by the photoionization of primary CH_3_. radicals at 19.23 eV, but C^+^ can be formed this way only if the methyl radical is internally excited before ionization (the appearance energies from ground-state methyl radical are 9.8 eV for methyl ion, 15.1 eV for CH_2_^+^ +H, 19.8 eV for CH^+^ +2H, 15.3 eV for CH^+^ +H_2_, 19.4 eV for C^+^ +H_2_ + H^28^).

In Fig. 2 (top) we show the relative ion yields in the delay range 0–5 ps. The inset shows the trends in the extended delay range 0–200 ps. A large relative increase is evident in the production of the CH_*x*_^+^ ion family. After 1–1.5 ps, the ion yields are substantially stable, up to 200 ps.

In Fig. 2 (bottom) we show the OH^+^ and the parent peak ion yields as a function of delay in the range 0–5 ps, and in the inset we show those in the extended delay range 0–200 ps. The OH^+^ ion (appearance energy of OH^.^ radical 13 eV^28^) shows a very small increase in abundance during the first 2 ps after the pump, and then at longer delays shows a growing trend. Since the FEL photon energy is amply sufficient to ionize the OH radical, the observed trend is possibly compatible with the observation^18^ that the neutral OH fragment is a major product after some hundreds of ps. Our results, to be discussed later in the paper, show that OH fragments are generated from the vibrationally hot ground state after internal conversion from S_1_ or intersystem crossing from T_1_. The parent peak shows a continuous decrease over 1 ps, and then stabilizes.

### Photoelectron spectra

**Fig. 3:**
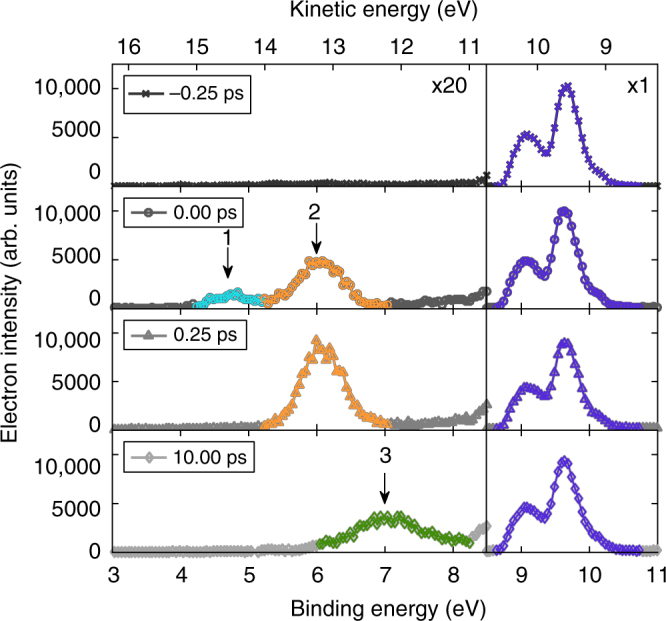
Valence photoelectron spectra for a series of pump-probe delays

Representative photoelectron spectra are shown in Fig. 3, which is segmented into two parts. The binding energy range from 8.5 to 11 eV (electron kinetic energy range from 10.5 to 8 eV) shows photoionization of ground-state molecules, while the binding energy range from 3 to 8 eV shows photoionization of excited molecules. The two strongest bands, at 9.0 and 9.6 eV binding energy, are due to the enol form of the unexcited molecule^14,15^. The enol structure is the stable form at room temperature and the two main peaks correspond to molecular orbitals distributed on the π system and the oxygen lone pairs^14^. There are no significant changes in the valence photoelectron spectra of the ground state as a function of pump-probe delay. This observation rules out one of the possible photochemical processes, namely 261 nm photon-induced isomerization from the enol to the keto form (followed by relaxation back to the ground state): if that were to occur there would be two spectral features growing, corresponding to the + and − combinations of the oxygen lone pair orbitals in the keto form and including the band at 10.2 eV, while the first peak corresponding to the π system of the enol form should be depleted^14,15^.

While the photoelectron spectra corresponding to ionization of the ground state do not show any significant effect due to photoexcitation, the situation is very different for the low-binding energy region, where the spectral structures correspond to the ionization of excited species formed by the UV pump at 261 nm. We can identify three peaks in this region, at apparent binding energies of 4.64, 6.04, and 7.14 eV, each showing a different time evolution in the delay range 0–2 ps. To assign the three observed spectral features to the related excited states we have performed ab initio static and surface-hopping dynamics calculations. The relevant molecular structures optimized at the MS-CASPT2[10,10] level^29,30^ are shown in Supplementary Fig. 1 (for Cartesian coordinates see Supplementary Table 1), while the corresponding excitation energies and mass-weighted distances from the reference ground state geometry (S_0min_) are given in Supplementary Tables 2 and 3. Ionization energies (IE) also calculated at the MS-CASPT2[10,10] level are reported in Table 1.

**Table 1:** Experimental and theoretical ionization energies (IE) of acetylacetone to the first ionic state (in eV) for the investigated transitions

Experiment	4.64	6.04			7.14	
Transition	D_0_ ← S_2_	D_1_ ← S_1_	D_0_ ← T_1_	D_1_ ← T_2_	D_0_ ← T_1_	D_1_ ← S_1_
at the geometry of	S_0min_	S_1min_	S_1min_	S_1min_	T_1min_	T_1min_
Static	4.43	5.70	5.78	5.77	6.77	6.70
Wigner		5.95 ± 0.39	5.87 ± 0.24	5.91 ± 0.24	6.78 ± 0.54	–
Dynamics	4.45 ± 0.39		5.81 ± 0.42		6.89 ± 0.66	

D_0_ corresponds to the first ionic state for the configurations (ππ*) (S_2_ and T_1_) and D_1_ corresponds to the first ionic state for the configuration (nπ*) (S_1_ and T_2_). Static denotes IE computed at selected geometries, Wigner denotes averaged IE (and standard deviations) computed for an ensemble of nuclear geometries sampled from the Wigner distribution at the given geometry. Dynamics denotes averaged IE (and standard deviations) computed at geometries extracted from surface hopping dynamics simulations. For details see Computational methods

The calculated IE (D_0_−S_2_) = 4.43 eV from the bright S_2_ state at the S_0_ minimum (S_0min_) geometry is in good agreement with the observed 4.64 eV energy of the lowest-lying peak (light blue curve). The equilibrium geometry of the S_1_ state, S_1min_, is geometrically close to the ground state minimum S_0min_ (Supplementary Table 3). At this geometry, the calculated ionization energy of 5.70 eV corresponds to the peak at 6.04 eV in the spectrum (orange curve). However, the peak at 6.04 eV has a more complex origin because at the S_1min_ geometry three electronic states, the singlet S_1_ (nπ*) and triplets T_2_ (nπ*) and T_1_ (ππ*) are separated by only 0.1 eV. As the ground state of the cation is accidentally degenerate at this geometry with two states separated by only 0.02 eV, the IEs of all three states match the peak at 6.04 eV (Table 1).

As for the third peak at 7.14 eV, the IE calculated for the minimum energy geometry of the T_1_(ππ*) state of 6.77 eV is in agreement with the experimental value (green curve). The minimum, T_1min_, is highly deformed relative to the planar ground-state structure. At this geometry, the IE of the S_1_ state (6.70 eV) also matches the experimental peak (Table 1). Therefore, the peaks at 6.04 and 7.14 eV may originate from different electronic states and can be attributed to ionizations from planar and distorted structures, respectively.

### Time evolution

**Fig. 4:**
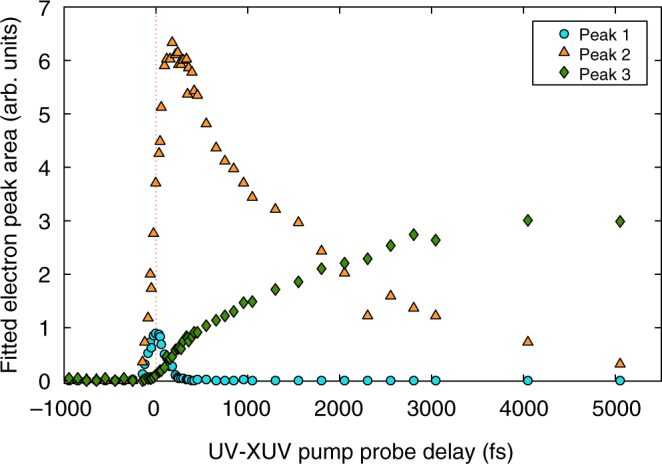
Experimental peak areas as a function of pump-probe delay. Given for peak 1 (4.64 eV), peak 2 (6.04 eV), and peak 3 (7.14 eV)

The transition from the S_2_ (ππ*) singlet state to the S_1_ (nπ*) state, with possible crossing to the T_2_ (nπ*) and T_1_ (ππ*) manifolds, and finally molecular deformation and relaxation to the T_1_ (ππ*) state, are clearly reflected in the evolution over time of the three spectral features (Fig. 3) in this region corresponding to the ionization of the excited acetylacetone. Figure 4 shows fitted experimental peak areas intensities as a function of pump-probe delay time. The signal from the S_2_ state (light blue curve) decreases rapidly and disappears at about 50 fs delay, the signal attributed to S_1_/T_2_/T_1_ states arising from planar structures increases rapidly up to about 500 fs and then decreases, and the peak related to the T_1_ state and non-planar structures increases up to about 3 ps and then becomes stable.

**Fig. 5:**
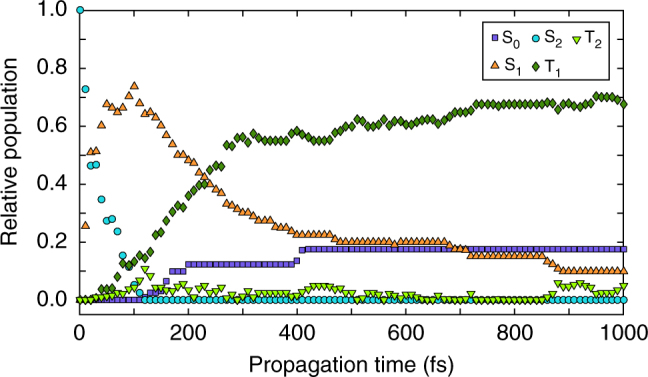
Average population of the adiabatic states. Given for the electronic ground state (S_0_), the two lowest singlet states, S_2_ (ππ*) and S_1_ (nπ*), and two triplet states, T_2_ (nπ*) and T_1_ (ππ*), obtained with CASSCF-based nonadiabatic dynamics simulations

To interpret the experimental trends, the calculated average population of all electronic states populated during the dynamics is shown in Fig. 5. Note that the evolution of the electronic state populations shown in Fig. 5 and the dynamics of the three electron peaks, shown in Fig. 4, are not directly comparable quantities, as three electronic states match the electronic peak centered at 6.04 eV (see Discussion section below).

## Discussion

The 261 nm excitation promotes the molecule to the S_2_ (ππ*) state. On the S_2_ (ππ*) surface, the system initially evolves by elongation of the carbonyl C=O bond. The energy stabilization of the S_2_ state along the C=O stretching coordinate leads to the CIs with the S_1_ (nπ*) state. From Fig. 5 it is evident that the S_2_ (ππ*)/S_1_ (nπ*) CI seam is reached early in the dynamics as a complete population transfer to the S_1_ (nπ*) state takes place within 100 fs, in agreement with the fast disappearance of the experimental peak at 4.64 eV.

Once in the S_1_ (nπ*) state, the trajectories split into three groups. Most trajectories (60%) reach the T_1_ (ππ*) state through ultrafast S_1_ (nπ*)/T_2_ (nπ*) crossing which is immediately followed by internal conversion to T_1_ (ππ*). T_2_ (nπ*) is a doorway state and its population never reaches more than 10% (see Fig. 5). A smaller portion (15%) quickly passes from the S_1_ (nπ*) state to the ground state, but this pathway is effectively closed after 400 fs. The remainder of trajectories stayed trapped in the S_1_ (nπ*) state.

To make a direct comparison with the time evolution of the experimental peaks we computed the IEs (Table 1, Dynamics). Based on the results of dynamics simulations, IEs have been computed in time steps of 50 fs and averaged over all nonadiabatic trajectories residing in a given electronic state. From the IEs presented in Table 1, one notices that, as long as the molecular ring is almost planar, the transfer of population S_1_ → T_2_ → T_1_ will not be observed in the experimental spectrum as the third peak at 7.14 eV. This peak arises unequivocally from the T_1_ state when the molecule deplanarizes.

However, in the dynamics simulations, the rise of the peak at IE = 6.89 eV is exceedingly fast and occurs rapidly after crossing to the T_1_ state. This suggests that either intersystem crossing is overestimated, causing excessively fast population transfer to the T_1_ state, or that the molecule deforms too easily on the T_1_ surface. By comparing CASSCF and CASPT2 SOC and energy differences between S_1_ and the triplet states, we could rule out the first possibility, since we found no indication that the time scale of intersystem crossing is underestimated in the CASSCF-based dynamics. To investigate the second possibility, we computed the CASSCF and CASPT2 energy differences between T_1min_ and a planar transition state, i.e., T_1ts_, that connects the two equivalent T_1min_ minima. Supplementary Fig. 2 shows that at the CASSCF level the energy difference is overestimated (0.37 eV) compared to the reference CASPT2 result (0.12 eV) causing indeed a fast deplanarization and early appearance of the peak at 7.14 eV in the dynamics.

Additional information on the evolution of the system can be extracted from the ion spectra. To identify the electronic states from which fragmentation takes place, we computed the reaction paths for CH_3_ and OH dissociation in the T_1_ (ππ*) and S_1_ (nπ*) states (Supplementary Figs. 3 and 4). In the relaxed scans computed at the MS-CASPT2[10,10] level, the barriers to dissociation should be compared to the S_2_ (ππ*) excitation energy of 4.99 eV, which is the total amount of energy available to the system after photoexcitation. The potential energy profiles obtained on the T_1_ state show that the dissociation of the methyl group starting from the planar T_1_ (ππ*) minimum is possible. The barrier of *E* = 4.54 eV is significantly lower than either the one on the S_1_ (nπ*) surface (~5.0 eV) or the one for the non-planar path on the T_1_ (ππ*) surface (>5.0 eV). In contrast to the formation of methyl fragments, dissociation to form the OH radicals is not feasible from either of the excited states, implying that OH fragments are generated from the vibrationally hot ground state after internal conversion from S_1_ or intersystem crossing from T_1_. Reaction path calculations on the ground electronic state show that dissociations to both OH and CH_3_, with barriers of 4.02 and 3.67 eV, respectively, are possible on this surface.

The occurrence of CH_3_ fragmentation only along the planar path on the T_1_ (ππ*) surface suggests ultrafast population transfer to this state. In the experiment, there is a small difference in the rise of the ion (Fig. 2) and the 7.14 eV photoelectron curves (Fig. 3, green line). The first reaches a plateau at ~1 ps whereas the second, arising from non-planar geometries in the T_1_ (ππ*) state, becomes stable only after 2 ps. Thus, combined ion and photoelectron spectra, as well as static and dynamics calculations, hint at an S_1_/T_1_ intersystem crossing in acetylacetone taking place on the picosecond or even subpicosecond time scale and leading to early appearance of CH_3_ fragments. However, the possibility that CH_3_ fragmentation occurs in the ground electronic state after ultrafast internal conversion from the S_1_ state cannot be ruled out.

**Fig. 6:**
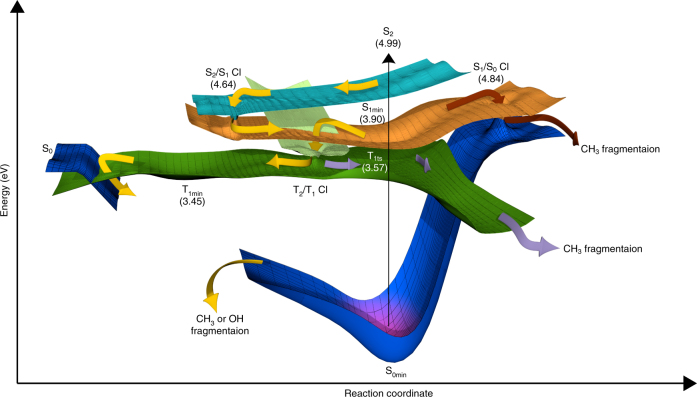
A schematic overview of the relaxation mechanism of acetylacetone. The ground state S_0_ (darker blue), two singlet S_2_ (ππ*) (light blue) and S_1_ (nπ*) (orange), and two triplet T_2_ (nπ*) (light green) and T_1_ (ππ*) (green) states are shown. Excited state minima and minimum energy CIs (MECI) are indicated. Relative energies with respect to the electronic ground state minimum (S_0min_) are given. For details see Supplementary Tables 1–3

The nature and time evolution of all states involved in the photodynamics are pictorially illustrated in Fig. 6 (see also Supplementary Fig. 6), which is to be considered a graphic summary of the behavior of the system. Schematically we illustrate the potential surfaces of the ground state and the two lowest singlet and triplet surfaces, as well as their crossing points, CIs and possible fragmentation pathways. Following the ultrafast internal conversion from the S_2_ (ππ*) state (light blue) to the S_1_ (nπ*) state (orange), three scenarios are possible:

(i) Internal conversion from S_1_ (nπ*) to S_0_ involves passing through a high-lying seam of CIs (S_1_/S_0_ CI at 4.84 eV) (Fig. 5, brown arrows). The relative inaccessibility of this pathway is consistent with the slow disappearance of the S_1 _(nπ*) peak at 6.04 eV.
(ii) The dominant deactivation pathway leads to the population of the T_1_ (ππ*) (green) state via the doorway state T_2_ (nπ*) (light green). The high efficiency of intersystem crossing is due to the fact that for planar geometries close to S_1min_ the energy gap between the S_1_ (nπ*), T_2_ (nπ*) and T_1_ (ππ*) states is very small. Norrish type-I methyl fragmentation is initiated upon crossing to the T_1_ state along a planar reaction path (Fig. 6, violet arrows). The pathway is consistent with the observed fast increase in the ion yield of the CH_*x*_^+^ fragments (Fig. 2).
(iii) The system undergoes a roaming dynamics on the flat portion of the T_1_ (ππ*) potential energy surface. On a longer time scale, geometry deformations on the T_1_ state facilitate crossing to the electronic ground state from which both CH_3_ and OH fragmentations are possible (Fig. 6, yellow arrows).

For comparison with previously reported results on the same system with a similar UV excitation as pump and different probe tools, we can make the following observations: In ref. ^18^ it was reported that the production of the OH fragment is related to the onset of formation of the T_1_ state, with a time constant of 247 ps. However, from our data set we can clearly see that the formation of the T_1_ state occurs much earlier, and it is achieved within few ps. This discrepancy is to be attributed to the fact that in ref. ^18^ electron diffraction was used to follow the evolution of the system, which cannot detect the small geometry changes between all excited states. Therefore, the onset of formation of OH should be attributed to the relaxation to the vibrationally hot ground state (see above Discussion). In ref. ^19^ the evolution from S_2_ to S_1_ was investigated with 266 nm pump–800 nm multiphoton probe in the delay range up to a few ps and low-kinetic energy electron spectra summed over several delays were shown. Ion yields were also recorded for some of the fragments, but not for the CH_*x*_ family. A one-to-one spectral assignment for the various excited states as a function of pump-probe delay was not reported, and the transition to triplet states was not taken into consideration.

## Methods

### Experimental

The experiments were performed at the LDM beam line^24,25^ of the FERMI FEL^7^, Trieste, Italy. The pump laser, denoted SLU (Seed Laser for Users), was set at the 3rd harmonic (261 nm) of the Ti:Sa base laser, horizontal polarization. The FEL beam was set at the fourth harmonic (19.23 eV) of the seed laser^8^, thus *λ*_seed_ = 257.85 nm, *λ*_FEL_ = 51.57 nm. Horizontal polarization was used.

A highly-efficient, high-resolution magnetic bottle electron spectrometer with a retardation lens system of the Eland type^21–23^, similar to what was recently used at the AMO end station of the LCLS at Stanford, has been built and installed on LDM for the experiment. One of the latest development of our magnetic bottle technique allows detection of both electrons and ions at high collection efficiencies, retaining high energy resolution in particular for the electrons^22,23^, which was very important to match the superior performances of the FERMI light source^31^ (see Supplementary Note 1 for a more detailed description of the spectrometer).

### Data treatment

The raw electron/ion spectra were binned according to delay time and filtered so that shots with poor quality FEL light (low pulse energy) were rejected. The signal was summed and then normalized to the total FEL XUV energy in that bin as measured by the shot-to-shot energy detectors^32^. The electron time-of-flight spectra were then converted to kinetic energy using a variable width binning method, and a calibration derived from an ATI (above-threshold ionization) spectrum taken with the fundamental IR laser beam. The main peaks were referenced to the 2nd harmonic signal from ionization in He, which gives a constant spectral feature around 14 eV electron energy (see Supplementary Note 2 for more details).

### Computational static calculations

Excitation energies were computed by performing a state-averaged multiconfigurational self-consistent field (SA-MCSCF) calculation including four singlet and two triplet states. The active space was selected by treating ten valence electrons as active electrons distributed among ten orbitals. The orbitals involved in the active space at the ground state minimum energy geometry are shown in Supplementary Fig. 5. For ionization energies, a state-averaged calculation including two doublet states was performed using nine active electrons in ten orbitals. The energies were corrected by including the residual dynamical correlation contribution through multistate second order perturbation theory (MS-CASPT2)^29^. The cc-pVDZ basis set was used throughout the work. The Molcas^30^ package was used for all calculations.

Minimum energy CIs and singlet-triplet minimum crossing points were computed at the same MS-CASPT2[10,10] level using the sequential penalty constrained optimization method of Levine et al.^33^ with default initial values of *α* = 0.025 Hartree and *σ* = 3.5.

The harmonic-oscillator Wigner distribution for the nuclei was used to compute the ionization energies at the minima of the S_1_ and T_1_ states (Table 1) as well as the SOCs^34^. The computations were performed at the MS-CASPT2[10,10] level. When relaxing from the Franck–Condon region toward the minimum of the S_1_ state, the system gains ~1.1 eV of kinetic energy, which is redistributed into molecular vibration. Accordingly, the Wigner distributions ware computed for an effective temperature of 600 K.

Altogether 150 nuclear coordinates were sampled for each minimum.

Deactivation pathways obtained from dynamics simulations and ion yield measurements were explored at the MS-CASPT2[10, 10] level. Linear interpolation in internal coordinates was used to connect optimized structures such as excited state minima and transition states. The intermediate geometries on these linearly interpolated paths were not optimized (rigid scans). Constrained MS-CASPT2[10, 10] optimizations along the C-CH_3_ and C-OH stretching coordinates were performed to obtain relaxed scans of the potential energy for CH_3_ and OH dissociation.

### Computational dynamics calculations

Nonadiabatic molecular dynamics simulations have been performed using Tully’s fewest switches surface hopping algorithm^35^. Two sets of simulations have been performed. The first was based on the algebraic diagrammatic construction to second order (ADC(2)) method^36–39^ and was restricted to singlet surfaces. The second, which was based on the CASSCF method and employed the same CAS-[10, 10] space as the one used in the static calculations, allowed switching between potential energy surfaces of different multiplicity. For the latter the SHARC program was used^40–42^. In SHARC calculations both nonadiabatic and SOC were included. SOC were calculated among four singlet (S_0_-S_3_) and two triplet (T_1_, T_2_) states. The surface hopping procedure was performed using the so-called diagonal states, i.e., states obtained by a diagonalization of the total Hamiltonian which includes SOC. The required gradients of the diagonal states were computed from the spin-free ones^41^. To facilitate the interpretation of the results, the populations of the electronic states shown in Fig. 5 have been transformed to the adiabatic basis.

In total, 50 ADC(2) and 40 CASSCF trajectories have been launched from the S_2_(ππ*) state and propagated for 1 ps. Initial coordinates and velocities were generated from the vibrational ground state Wigner distribution which is the preferred sampling method for gas-phase systems of the size of acetylacetone^43^. Newton’s equations were integrated with time steps of τ1 = 0.5 fs whereas the time-dependent Schrödinger equation was integrated in time steps of τ2 = 5 ∙ 10–5 fs. The energy dependent decoherence scheme of Granucci and Persico with the default correction parameter of 0.1 Eh was employed in both types of simulations^44^.

ADC(2) dynamics predicted an ultrafast S_2_ → S_1_ → S_0_ deactivation along the C=O stretching and out-of-plane OH motion. CASSCF dynamics predicts a S_2_ → S_1_ → T_1_ → S_0_ decay that finds good agreement with the experiment.

### Data availability

The data sets generated during and/or analyzed during the current study are available upon reasonable request from the corresponding author (M.N.P.).

## Electronic supplementary material

Supplementary Information
