# Supplementary material for: Acetylacetone photodynamics at a seeded free-electron laser
Source: Nat Commun. 2018 Jan 4;9:63. doi: 10.1038/s41467-017-02478-0 (PMC5754354; doi:10.1038/s41467-017-02478-0)
Supplement: Supplementary file 1 — Supplementary Information [file 41467_2017_2478_MOESM1_ESM.pdf]

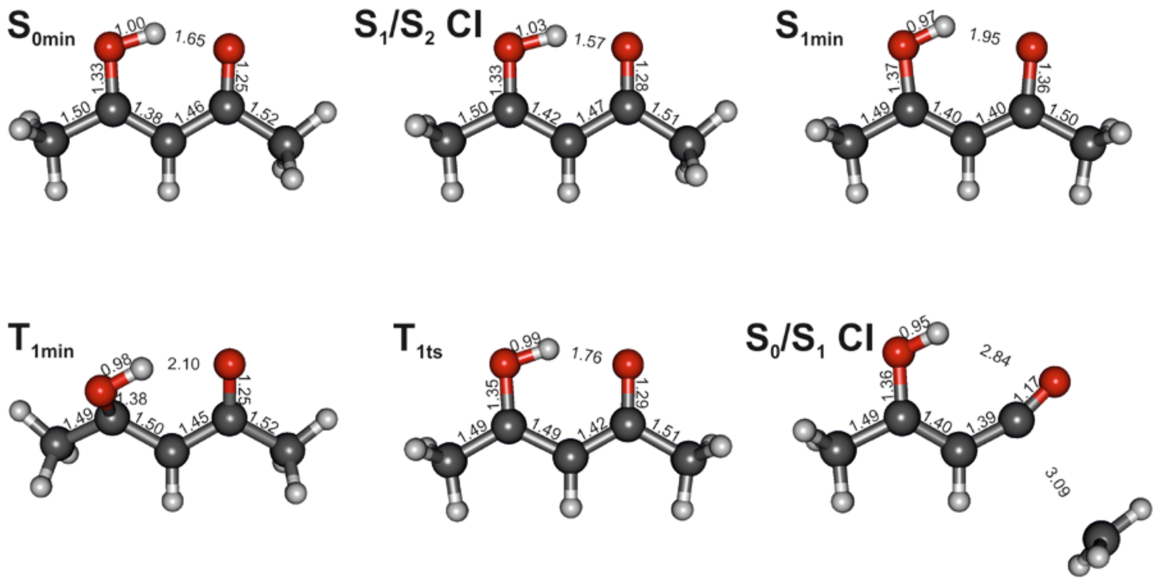

Supplementary Figure 1. Relevant molecular structures optimized at the MS-CASPT2[10,10] level. Distances are in Ångstroms. For Cartesian coordinates see Supplementary Table 1.

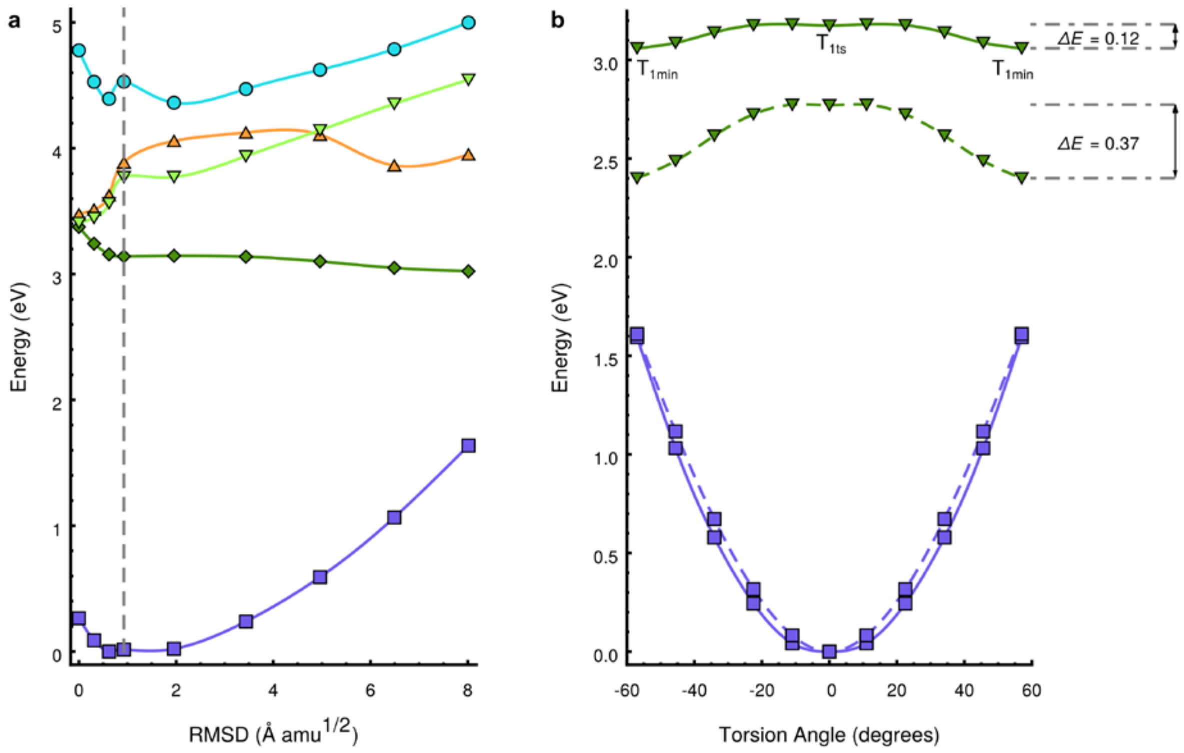

Supplementary Figure 2. a) Linearly interpolated path from S_{1min} (nπ*) to T_{1ts} (ππ*) and from T_{1ts} (ππ*) to T_{1min} (ππ*). MS-CASPT2 energy profiles of the ground electronic state (dark blue), the singlet states S_2 (ππ*) (light blue) and S_1 (nπ*) (orange) states and the triplet states T_1 (ππ*) (green) and T_2 (nπ*) (light-green) are shown. The vertical line indicates two interpolation regions. b) Comparison of MS-CASPT2 (full) and CASSCF (dashed) energy profiles along the linearly interpolated path from T_{1ts} (ππ*) to T_{1min} (ππ*). The electronic ground state (dark blue) and the triplet states T_1 (ππ*) (green) are shown. The energy difference between the transition state T_{1ts} (ππ*) and the minimum T_{1min} (ππ*) is only ΔE=0.12 eV at the MS-CASPT2 level and ΔE=0.37 eV at the CASSCF level. This difference contributes to the fast deplanarization of the system and the too early appearance of the peak at 7.14 eV in CASSCF-based dynamics.

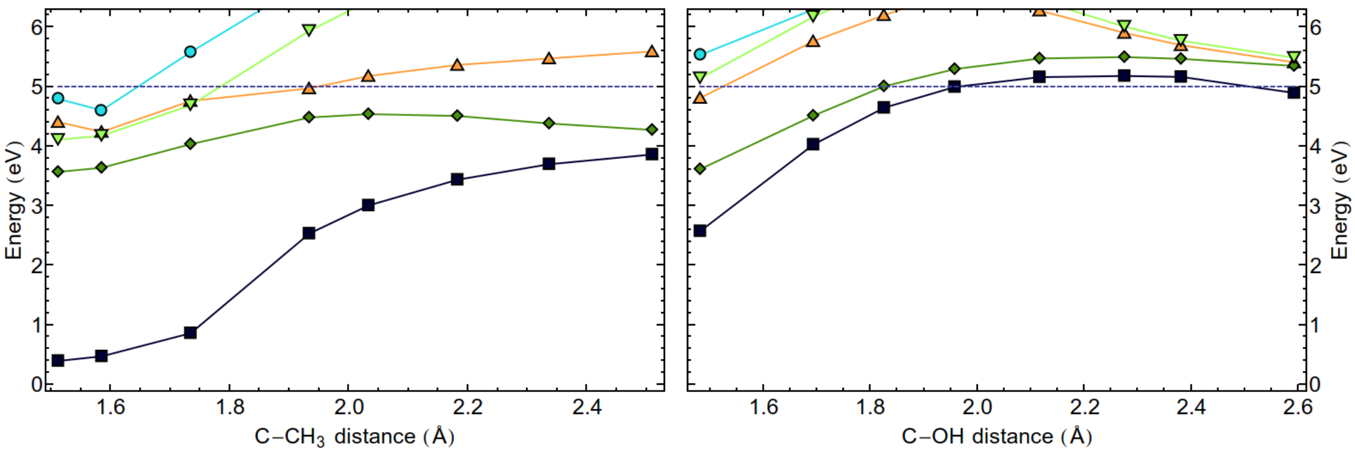

Supplementary Figure 3. Energy profiles for the generation of methyl (left) and OH (right) fragments in the T_1 ($\pi\pi^*$) state. MS-CASPT2[10,10] optimized reaction paths are shown. The ground state S_0 (dark blue), two singlet S_2 ($\pi\pi^*$) (light blue) and S_1 ($n\pi^*$) (orange) and two triplet T_2 ($n\pi^*$) (light green) and T_1 ($\pi\pi^*$) (green) states are shown. All energies (in eV) are given with respect to the ground state minimum energy. The planar T_{1ts} transition state is the initial structure for generation of CH_3 radicals. The bent minimum T_{1min} is the initial structure for generation of OH radicals. The energy difference between the two initial structures is only 0.12 eV. Gray dots indicate the vertical excitation energy of 4.99 eV which is the total amount of energy available to the system for the reaction. In the T_1 state, generation of methyl fragments is possible only along the planar reaction path (left). Generation of OH fragments is not possible (right).

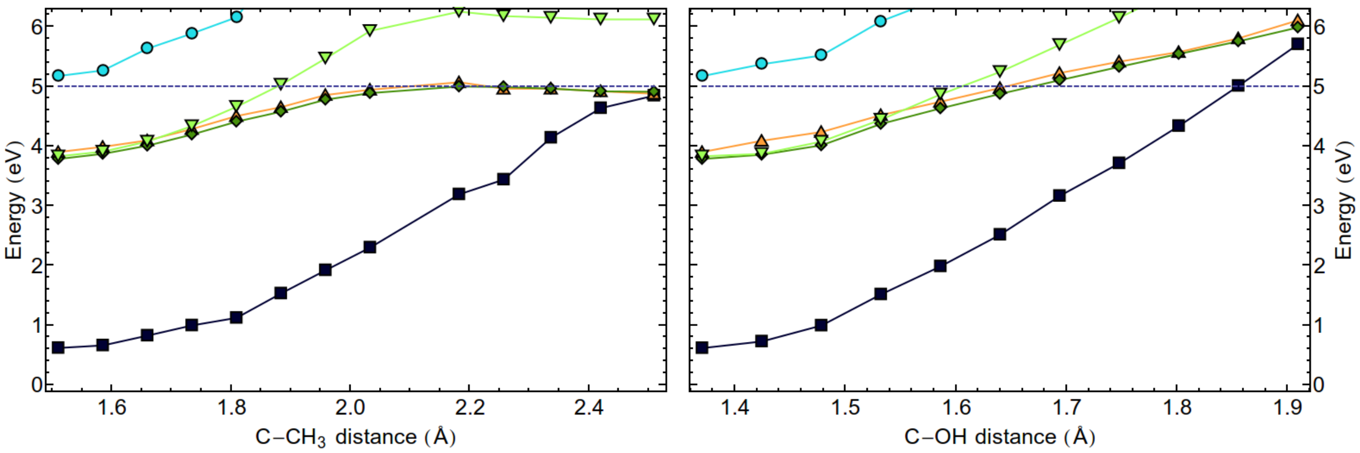

Supplementary Figure 4. Energy profiles along MS-CASPT2 optimized reaction paths for methyl (left) and OH (right) dissociation in the S_1 (nπ*) state. The ground state S_0 (dark blue), two singlet S_2 (ππ*) (light blue) and S_1 (nπ*) (orange) and two triplet T_2 (nπ*) (light green) and T_1 (ππ*) (green) states are shown. All energies (in eV) are given with respect to the ground state minimum energy. The initial geometry is the S_1 minimum. Gray dots indicate the vertical excitation energy of 4.99 eV. C-C bond dissociation to generate methyl fragments is barely possible in the S_1 state (left) whereas generation of OH fragments is not possible from the S_1 state (right).

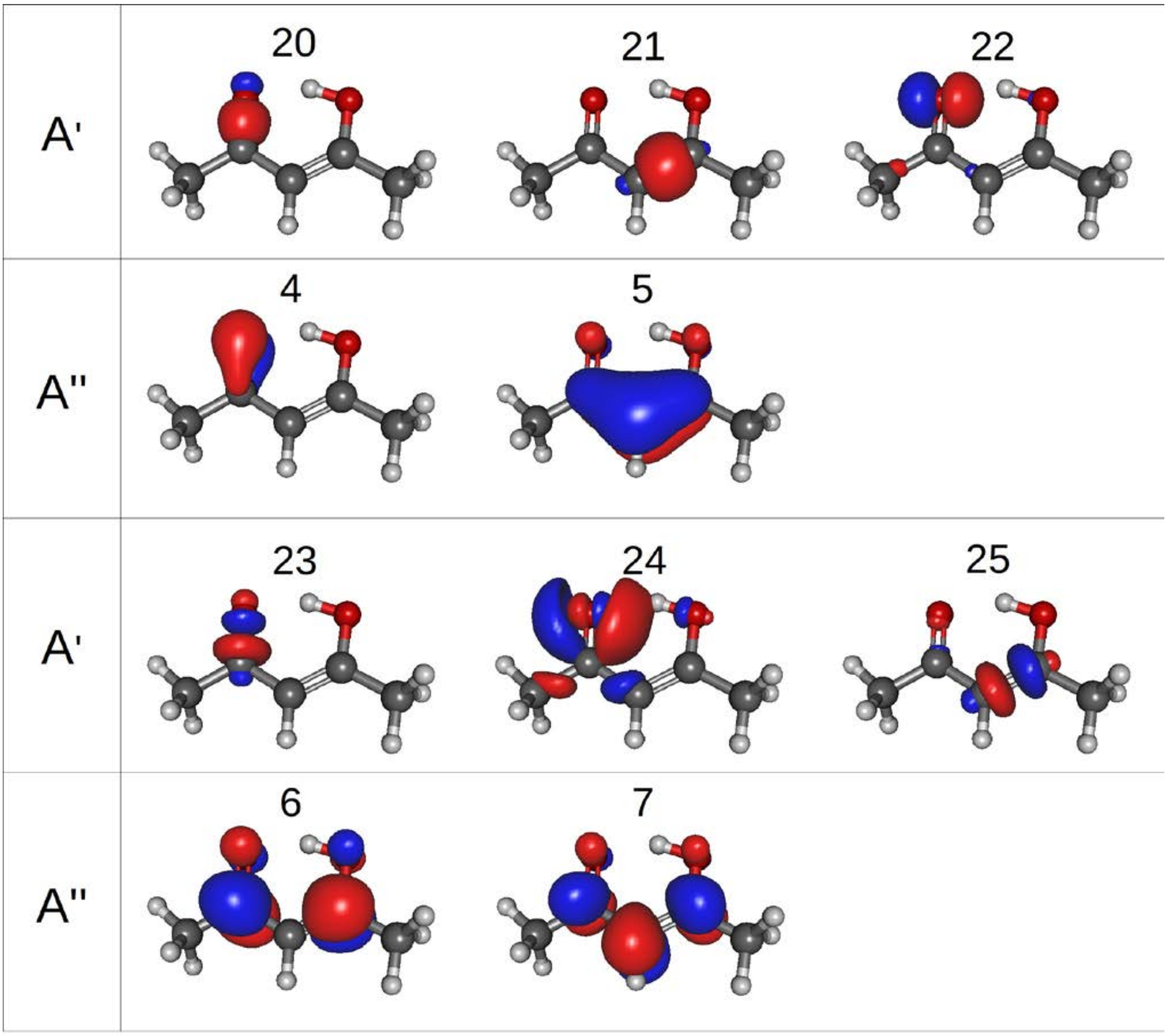

Supplementary Figure 5. The orbitals involved in the active space at the ground state minimum energy geometry (C_s symmetry). Ten valence electrons are distributed in ten orbitals: six A' and 4 A''. The 5 occupied orbitals are 20-22 A' and 4-5 A'' and the 5 virtual are 23-25 A' and 6-7 A''.

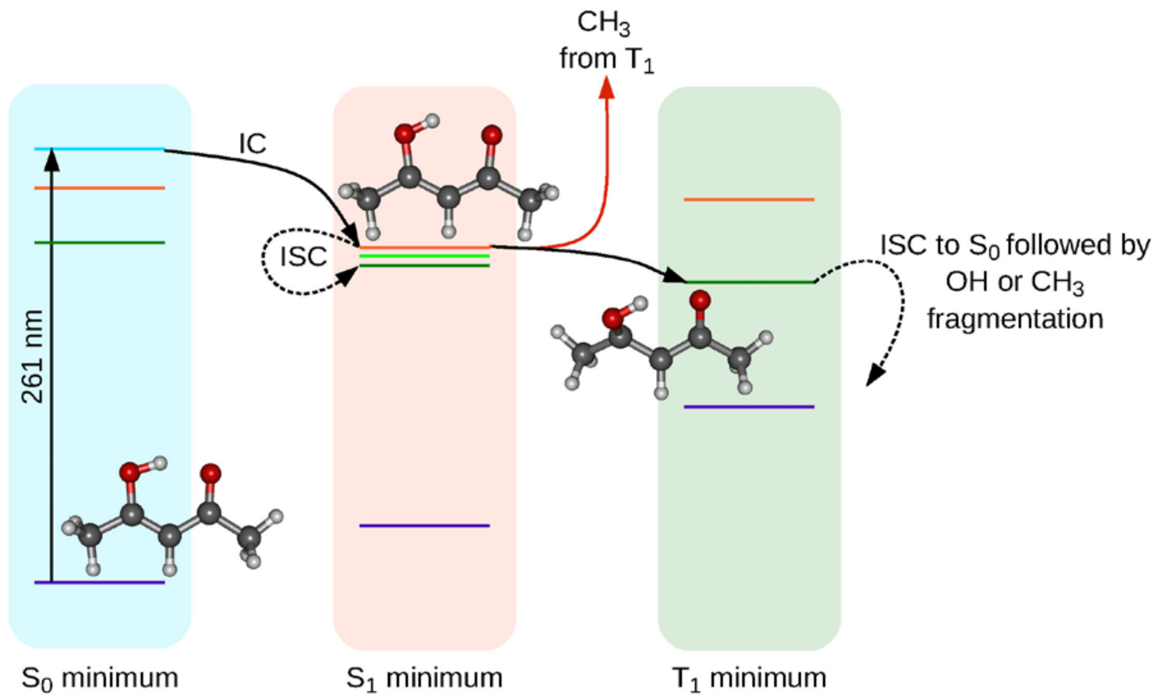

Supplementary Figure 6. Relaxation mechanism of acetylacetone as obtained based on experimental and computational results. IC stands for internal conversion between states of the same spin and geometry relaxation within a single state (full arrows), while ISC stands for inter system crossings (dashed arrows). Energies of the ground state S_0 (violet), two singlet S_2 (ππ*) (light blue) and S_1 (nπ*) (orange), and two triplet T_2 (nπ*) (light green) and T_1 (ππ*) (green) states are given with respect to the minimum of the electronic ground state, S_{0min}. The blue, red and green areas mark the states/geometries responsible for the photoelectron spectrum peaks at 4.64, 6.04 and 7.14 eV, respectively. For details, see Supplementary Tables 1-3.

	S_0			S_{1min}			S_0/S_1 CI		
C	-2.552	-0.825	-0.001	-2.609	-0.806	0.000	-3.415	-1.257	-0.177
C	-1.254	-0.048	0.001	-1.268	-0.130	0.000	-0.928	0.552	0.148
O	-1.271	1.198	0.001	-1.350	1.230	0.001	-1.439	1.607	0.131
C	-0.002	-0.804	0.001	-0.020	-0.765	0.000	0.020	-0.469	0.140
C	1.205	-0.140	0.000	1.239	-0.144	-0.001	1.391	-0.250	-0.055
O	1.296	1.191	-0.001	1.422	1.217	-0.001	1.917	0.994	-0.218
C	2.537	-0.826	0.000	2.537	-0.882	0.001	2.425	-1.318	0.102
H	-0.019	-1.894	0.001	-0.042	-1.857	-0.001	-0.346	-1.474	0.270
H	-3.403	-0.131	0.016	-3.198	-0.520	0.889	-4.033	-0.372	-0.094
H	-2.606	-1.461	-0.900	-3.194	-0.525	-0.894	-3.252	-1.682	-1.158
H	-2.593	-1.491	0.877	-2.467	-1.896	0.003	-3.317	-1.899	0.689
H	2.428	-1.919	-0.003	2.373	-1.970	-0.010	1.970	-2.308	0.057
H	3.110	-0.516	0.890	3.133	-0.624	0.895	2.943	-1.223	1.063
H	3.112	-0.512	-0.887	3.144	-0.607	-0.881	3.178	-1.239	-0.687
H	0.346	1.504	0.000	0.553	1.650	0.000	1.300	1.581	-0.630
	T_{1ts}			T_{1min}			S_0/T_1 crossing		
C	-2.606	-0.786	-0.144	-2.613	-0.815	0.001	-2.607	-0.608	-0.504
C	-1.302	-0.038	0.049	-1.293	-0.077	-0.002	-1.354	0.012	0.094
O	-1.280	1.160	0.383	-1.294	1.210	-0.009	-1.400	0.918	0.933
C	-0.048	-0.734	-0.198	-0.059	-0.780	0.007	-0.074	-0.495	-0.382
C	1.250	-0.079	0.172	1.279	-0.132	0.007	1.233	0.014	0.077
O	1.428	1.145	-0.427	1.370	1.215	0.007	1.803	0.931	-0.757
C	2.493	-0.903	0.252	2.566	-0.879	-0.010	2.263	-0.930	0.635
H	-0.056	-1.648	-0.811	-0.072	-1.874	0.018	-0.071	-1.188	-1.237
H	-3.448	-0.120	0.090	-3.204	-0.516	0.883	-3.487	-0.151	-0.029
H	-2.693	-1.137	-1.185	-3.197	-0.536	-0.892	-2.619	-0.418	-1.592
H	-2.641	-1.673	0.510	-2.482	-1.908	0.013	-2.593	-1.698	-0.333
H	2.339	-1.775	0.907	2.394	-1.967	-0.017	1.877	-1.564	1.456
H	3.323	-0.298	0.651	3.185	-0.633	0.874	3.119	-0.360	1.044
H	2.798	-1.277	-0.748	3.170	-0.619	-0.901	2.670	-1.605	-0.149
H	0.573	1.598	-0.285	0.435	1.535	0.004	1.126	1.561	-1.019

Supplementary Table 1. Cartesian coordinates of key molecular structures in Ångstroms. All excited state structures were optimized at the MS-CASPT2[10,10] level.

	S_{0min}	S_1/S_2 CI	S_{1min}	S_0/S_1 CI	T_{1ts}	T_{1min}	S_0/T_1 crossing
S_0	0,00	0.11	0.69	4.84	0.39	2.00	3.55
S_1	4.55	4.64	3.90	4.89	4.40	4.40	3.89
S_2	5.01	4.69	5.20	7.74	4.79	5.47	5.34
T_1	3.93	3.76	3.80	4.94	3.57	3.46	3.50
T_2	4.25	4.28	3.83	5.44	4.11	5.01	5.08

Supplementary Table 2. MS-CASPT2[10,10] excitation energies (in eV) to the two lowest singlet and triplet states computed at key geometries (columns) along the acetylacetone decay paths. The energies are given with respect to the minimum of the electronic ground state.

	S_{1min}(nπ*)	T_{1min}(ππ*)	T_{1ts}(ππ*)	S_1(nπ*) /S_2(ππ*) CI	S_0/S_1(nπ*) CI
S_{0min}	0.835	3.178	0.581	0.319	5.649
S_{1min}		3.120	0.519	0.942	5.325

Supplementary Table 3. Mass weighted root-mean square distances (mwRMSD) in Å a.m.u.$^{1/2}$ between relevant geometries on the acetylacetone decay paths. All structures are optimized at the MS-CASPT2[10,10] level. mwRMSD are obtained after maximizing the overlap between the two geometries. The relatively small mwRMSD between the Franck-Condon geometry, S_{0min}, and S_1 (nπ*) /S_2 (ππ*) CI and T_{1ts} (ππ*) indicates that these structures are encountered early in the dynamics, while the relatively large mwRMSD between S_{0min} and T_{1min} (ππ*) points to substantial structural deformations. The latter implies that T_{1min} (ππ*) is likely to be encountered later in the dynamics. Cartesian coordinates of all structures are given in the Supplementary Table 1.

Supplementary Note 1

The spectrometer is a magnetic bottle type electron spectrometer, chosen for its unparalleled collection efficiency combined with its good energy resolution over a broad range of electron kinetic energies. The operating principle is as follows: by using a combination of a strong and weak magnetic fields, the electrons, which are initially emitted in all directions, have their trajectories parallelized in the direction of decreasing magnetic field[1], ensuring that almost all electrons are directed towards the detector, providing a collection solid angle of 4π sterad, and collection efficiencies of 50-60% taking account of detector efficiencies. The strong magnetic field is created using a combination of a cylindrical neodymium permanent magnet and a conical, soft-iron pole cap to concentrate the magnetic field to a ~1 T field strength at its tip. The magnet assembly is encased in a cylindrical aluminum housing so that any applied electrostatic fields are more homogeneous across the interaction region.

The weak field is supplied using a solenoid of turn density = 500 turns/m, which extends from ~90 mm from the interaction region along the entire length of the 2 m long flight-tube to the 40 mm diameter micro-channel plate detector placed at the exit of the flight tube. A current of 0.5-2.0 A is sufficient to generate a magnetic field of a few mT. At the entrance to the flight-tube, a set of four lenses with an aperture of 20 mm and a spacing of 5 mm are used to apply a small potential across the interaction region to ensure electrons close to zero kinetic energy arrive within a reasonable flight time. Applying a potential to the final lens and the flight tube allows the electrons to be retarded by an arbitrary energy before entering the flight tube, which gives improved resolution in the higher kinetic energy regions of the spectrum at the expense of detection capability for any electrons with kinetic energy lower than this retardation potential.

While the spectrometer configuration is optimized for electron detection, ion detection is also possible. By applying negative electrical potentials of the order of a few kV to both the magnet and the three lenses, an approximation of a two-field Wiley-McLaren mass spectrometer configuration can be achieved. The spectrometer is able to resolve mass differences Δm of ~1/20 amu for masses in the 50-100 amu range (and hence a resolving power $m/\Delta m$ of ~1-2000), which is sufficiently accurate for identification of the different atomic and molecular fragment species created by XUV fragmentation.

Electrons and ions that hit the micro-channel plate detector produce an electrical pulse which is decoupled from the plates using an impedance matched anode. These signals are sent to the FERMI-LDM data acquisition system, consisting

of a CAEN VX1751 digitizer which converts the analogue waveforms to a digital form with time resolution of 1 ns and 10 bit dynamic range over the entire 1000 mV range. These waveforms are then stored in a Hierarchical Data Format (HDF)[2] for later analysis alongside correlated data such as pulse energy, bunch number and other experimental parameters.

Supplementary Note 2

Analysis of the acquired data was performed in several steps. Firstly, the data was filtered so that shots which contain corrupt data or where the FEL/optical pulse energy fell outside acceptable bounds (due to fluctuation of pulse energy) were eliminated. Secondly, the waveforms were binned according to the UV-FEL pump-probe nominal delay value associated with each shot, and then summed to generate an average electron/ion spectrum for each delay bin. These spectra were then scaled according to the total amount of FEL energy measured for all selected shots sorted into a particular delay bin, which is necessary to account for the possibility of fewer viable shots in each bin and also long term drifts of the FEL pulse energy that may result in variations in the total particle yield. Each of these spectra was converted from a time-of-flight spectrum to an energy spectrum by rebinning the normalised waveform into equal width energy bins using the formula $t = t_0 + C/\sqrt{(KE-E_0)}$, where KE is the electron kinetic energy, t is the time of flight and t_0, C and E_0 are calibration constants, with t_0 representing the digitizer time corresponding to the light arrival time, C is the electron path length scaling factor, and E_0 is the total potential the electrons are accelerated or retarded by before entering the flight tube. Electron peak areas in Fig. 4 were calculated using a nonlinear fitting algorithm using fixed position Gaussians for the spectra at each delay.

Supplementary References

[1] Kruit, P. & Read, F.H., Magnetic field paralleliser for 2π electron-spectrometer and electron-image magnifier, *J. Phys. E. Sci. Inst*. **16**, 313-324 (1983)
[2] www.hdfgroup.org
